# Volunteer-led behavioural activation to reduce depression in residential care: a feasibility study

**DOI:** 10.1186/s40814-020-00640-y

**Published:** 2020-07-07

**Authors:** Christina Bryant, Lydia Brown, Meg Polacsek, Frances Batchelor, Hannah Capon, Briony Dow

**Affiliations:** 1 Melbourne School of Psychological Sciences, University of Melbourne, 12th Floor, Redmond Barry Building, Parkville, VIC Australia; 2 North Eastern Rehabilitation Centre, Healthscope Hospitals, Melbourne, VIC Australia; 3 National Ageing Research Institute, Parkville, VIC Australia; 4 School of Physiotherapy, University of Melbourne, Parkville, VIC Australia; 5 School of Population and Global Health, University of Melbourne, Parkville, VIC Australia

**Keywords:** Behavioural activation, Depression, Residential aged care, Well-being

## Abstract

**Objectives:**

Symptoms of depression are highly prevalent and under-treated in residential aged care facilities. Behavioural activation is a simple, cost-effective psychosocial intervention that might be appropriate to help reduce depression and improve well-being in this setting. The purpose of this study was to investigate the feasibility and efficacy of an 8-week, volunteer-led behavioural activation intervention designed for depressed aged care residents.

**Method:**

This feasibility study employed a single-arm design, where outcomes were measured at baseline, post-intervention and 3-month follow-up. Aged care residents with depressive symptoms were invited to participate, and healthy volunteers were trained to deliver the intervention. Intervention feasibility was assessed on six a priori-determined domains. Depression, anxiety and flourishing were included as outcomes using intention-to-treat analysis.

**Result:**

Seventeen aged care residents with depressive symptoms and 13 volunteers were successfully recruited within the expected 6-month timeframe. Both residents and volunteers were satisfied with the intervention (7/8), and there was a high (87%) completion rate. The intervention was associated with a large and statistically significant reduction in resident depressive symptoms, *d* = − 1.14, with the effect increasing to *d* = 2.82 when comparing baseline to 3-month follow-up. Anxiety reduced from mild symptoms at baseline mean = *6*.17 (5.12) to the subclinical range post-intervention, mean = 3.53 (4.29) (*g* = 0.61, *p* = 0.03).

**Conclusion:**

This 8-week volunteer-led behavioural activation intervention was found to be feasible and acceptable to depressed aged care residents. The intervention was effective in ameliorating depression. A larger randomized controlled trial is warranted.

## Introduction

**Fig. 1:**
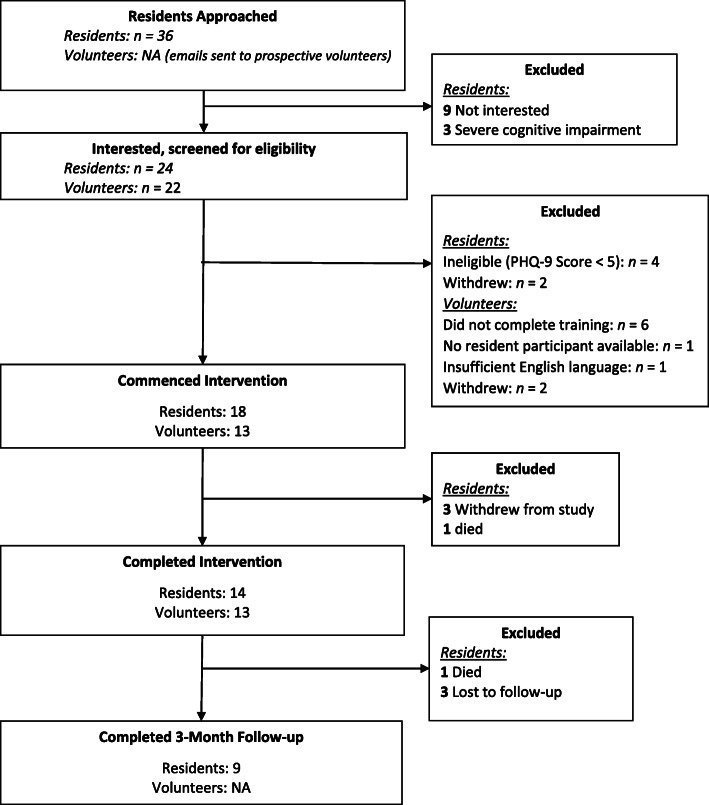
Flow chart of participants through the study

Depression is highly prevalent in care homes (also known as residential aged care facilities, nursing or care homes), is associated with serious consequences for physical and mental health, remains under-treated, and does not spontaneously remit [12, 35, 38]. The reported prevalence of depression in care homes ranges between 27% [39] and over 50% according to the most recent Australian government figures [1].

Despite a general consensus that psychological interventions, such as cognitive-behaviour therapy (CBT), are efficacious in the treatment of depression in older adults [8], most studies have been conducted with relatively young, physically well community-dwelling participants. In contrast, depressed residents in aged care facilities are likely to have physical co-morbidities, together with cognitive impairments [16, 41]. These characteristics make them less suitable as candidates for CBT, and the limited research on older adults’ preferences for psychological interventions suggests that adults over the age of 75 prefer more socially oriented approaches [25].

Behavioural activation (BA) is a brief structured treatment for depression that aims to increase engagement in pleasurable activities, with a focus on individualized problem solving geared towards overcoming the withdrawal from activities that commonly occurs in depression [5, 29]. Growing evidence supports the efficacy of BA as a treatment for depression in older adults [29] in both community and institutional settings. For several reasons, BA may be a very suitable intervention for older adults in residential care. Firstly, it is based on the relatively simple and intuitive principle that remaining active and engaged in activities is beneficial for mood [36], and can therefore be implemented flexibly, and with people with some degree of cognitive impairment [23]. The flexibility of BA means that relevant activities could include social interaction with volunteer visitors and physical activity, both of which are known to be associated with improvements in mood [24]. Secondly, as Polenick and Flora [31] reported, BA can be successfully implemented by non mental-health professionals [4, 31] and thus has the potential to be a relatively low cost, sustainable intervention that does not make heavy demands on aged care staff time, a factor known to be critically related to the effectiveness of psychological interventions in residential settings [6]. This is important because it can be difficult to engage staff in delivering interventions; staff report that they are constantly pressed for time, there is high staff turnover, and some may have low levels of training (Maas et al. 2002; Mavromaras et al. 2017). Therefore, if an intervention is to be feasible, cost-effective and sustainable, it needs to make minimal demands on staff time.

One way to reduce demands on staff is through the use of volunteers, who can be trained and supervised to assist with intervention delivery [11, 17]. McCurren et al. [22] developed and evaluated a programme in which volunteers were trained by a mental health nurse to deliver an intervention for aged care residents with depression measured with the GDS-30 [22]. The intervention included a detailed assessment of psychosocial needs and the provision of supportive counselling focused on discussing losses and emotions. Both residents and volunteers gave very favourable ratings to the volunteer programme, which resulted in resident depression scores declining by 40% in the intervention, but not the control group. Whilst volunteer depression scores were not measured, volunteers provided positive feedback that their participation was rewarding and worthwhile, suggesting that both residents and the volunteers themselves may benefit. This is in line with research supporting the value of volunteering for the well-being of those involved [28].

A further advantage of volunteers is that many older adults are unfamiliar with formal mental health treatment, but are more comfortable with paraprofessionals [17], perhaps because such models are more collaborative. Models for the use of volunteers vary, but one essential component is the use of an informal but clinically informed relationship to engage the older adult [17]. This relationship enables the volunteer and the older person to identify needs and goals and to work on behaviour change with the goal of alleviating depressive symptoms. This model is thus very compatible with the goals of BA.

Converging lines of evidence suggest that BA is a potentially effective intervention for the treatment of late-life depression in residential settings that can be delivered by non mental-health professionals. If adequately trained and supported, volunteers can be involved in intervention delivery, thus reducing the burden on paid staff [22]. To date, however, no study has evaluated the feasibility of trained volunteers implementing a structured BA intervention. Thus, the aim of this study was to train volunteers in BA and evaluate the feasibility and benefits of an 8-week BA intervention on levels of depression in adults living in residential care facilities. We were also interested to investigate whether participation in the programme was beneficial for the volunteers.

## Methods

### Design

This was a single-arm pre/post-intervention study design. Ethics approval for this study was sought and obtained from The University of Melbourne’s Human Ethics Committee (#1851320.1), and the trial was registered on the Australian New Zealand Clinical Trials Registry (approval # 1851320.1)

### Participants and recruitment

Three aged care facilities were approached, and two engaged in the programme. To be eligible for the study, resident participants were required to be full-time residents, aged 65 or over, who screened positive for the presence of depressive symptoms. Prospective participants were required to score ≥ 6 on the Patient Health Questionnaire-9 (PHQ-9), indicating at least mild symptoms of depression [20]. Participants were also required to have a conversational level of English. Exclusion criteria were the presence of medically diagnosed severe cognitive decline and psychosis. Potential resident participants were identified through discussion with aged care staff and approached by a member of the research team (MP or HC) and invited to participate in the study.

Volunteers were recruited from the volunteer database of Australia’s National Ageing Research Institute (NARI), as well as from the aged care provider’s pool of volunteers. Suitability included ability to commit to volunteer training and delivery of the 8-week intervention. Volunteers were required to meet the aged care provider volunteer criteria, including English language proficiency, being over the age of 18 and having a current Police Check.

All participants, including the volunteers, received a gift voucher from a retail group and a certificate of appreciation in acknowledgement of their time when they completed the study.

### Intervention

#### (i) Volunteer training

Volunteer participants attended two half-day training workshops where they participated in education on depression in older adults and the rationale for behavioural activation as a treatment for depression. Given the novelty of this study, there was no suitable pre-existing training manual. Rather, the training was developed primarily by author CB, an experienced clinical psychologist, drawing on BA principles [21] and literature relevant to BA for depression in older adults [23, 31]. The workshops included a combination of didactic and experiential learning using roleplays where volunteers were required to demonstrate key behavioural activation techniques. These included activity scheduling, mood monitoring and setting SMART goals, that is, goals that were specific, measurable, achievable, realistic and timely. Volunteers were also trained in risk monitoring for suicidality. Volunteers received feedback and guidance on their skills from a clinical psychologist (CB).

#### (ii) Behavioural activation intervention

The intervention consisted of 8 h of face-to-face contact provided in either 1 h or 30-min visits within 8 to 12 weeks. Volunteers were assigned to a resident by the facility’s lifestyle coordinator and a member of the research team. The volunteers then delivered the intervention at the resident’s aged care facility. Sessions followed a general structure, illustrated in Table 1 below. Throughout the intervention, volunteers received weekly phone calls from a trained member of the research team for support and supervision. A research team member was also available for support outside of weekly phone calls as needed.

**Table 1:** Overview of Intervention Session Structure

Session component	Description
1. Review Homework	Review resident’s activity schedule from the previous week. Discuss any problems or issues that arose whilst using the activity schedule.
2. Help resident to notice links between their behaviour and their mood	Using the activity schedule, volunteers inquired about any links that the resident noticed between their activities and mood.
3. Introduce and explore weekly theme	Volunteers were provided with resources for six domains *of pleasant activities: physical activity, relaxation, creativity, kindness, relationships and savouring. From week three to eight, volunteers were invited to introduce one of these themes to the resident, and explore a suitable activity relating to the theme that could be scheduled for the week ahead.
4. Activity scheduling for the week ahead	From week two onward, volunteers worked with the resident to plan pleasant activities for the week ahead using an activity scheduling sheet.

*These were identified by drawing on relevant literature and The Pleasant Events Schedule–Nursing Home Version [23]

### Measures

#### Primary outcome: Feasibility

Intervention feasibility was assessed on a priori-determined feasibility criteria recommended for use in feasibility studies [27]: recruitment, acceptability, attrition and safety. A measure of treatment fidelity was also included, to investigate the extent to which volunteers adhered to the activity scheduling protocol throughout the intervention. Descriptions of these criteria are outlined in Table 2.

**Table 2:** A priori feasibility outcome measures

Recruitment	Given that recruitment of older adults for psychology interventions is known to be slow and challenging (Moody et al. 2008), we expected that just 50% of residents who were approached would be interested in participating in the study. We deemed that approaching 40 prospective age-care residents, and recruiting approximately 20 residents (three to four residents per month) over a 6-month recruitment period (July 2018—Jaunary 2019) would be evidence of feasible recruitment.
Acceptability	On completion of the study, participants were asked to indicate their satisfaction. Qualitative feedback from participants was also collected at the conclusion of the final session.
Attrition	An intervention completion rate of ≥ 85% has been deemed acceptable in previous studies of older adults with physical conditions [3]. Therefore, a feasible attrition rate was determined to be 15% or less.
Safety	Behavioural activation is a low-risk intervention, with no known risks. However, any adverse psychological and/or physiological symptoms were recorded at each session. Volunteers were instructed to notify the research coordinator immediately following any adverse events.
Data collection	The research questionnaire was kept very brief, to minimize participant burden. We expected that data collection would not be problematic and that we would have minimal (< 5%) missing data.
Fidelity	The extent to which the volunteer facilitated activity scheduling with their assigned resident was taken as a measure of treatment fidelity. Activity scheduling was measured via volunteer submission of activity scheduling sheets and/or submission of notes outlining activity plans for the week ahead.

#### Secondary outcomes: Pre-post change

The following three measures were administered to residents and volunteers at baseline, on completion of the programme, at 8 weeks, and at 3-month follow-up.

##### Patient Health Questionnaire (PHQ-9)

The 9-item PHQ [20] is a validated measure of depressive symptom severity that is appropriate for both those with and those without physical health issues [20]. The PHQ has good psychometric properties [19], including with older adults [30]. Items are rated on a four-point Likert scale ranging from 0 to 3. One item pertaining to suicidality was omitted as it was thought to be too confronting for this cohort, and a final score was computed based on the mean of the remaining eight items multiplied by nine.

##### Generalized Anxiety Disorder Scale (GAD-7)

The GAD-7 [37] is a validated measure of symptoms of anxiety, with good psychometric properties, including with older adults [40]. It is frequently used in tandem with the PHQ-9 to generate a profile of anxiety and depressive symptoms.

##### Flourishing Scale

The Flourishing Scale [10] is an eight-item measure of eudaimonic well-being that has previously been used in studies of older adults [18] and validated in a sample that included over 1900 older adults [15].

### Residents only completed the De Morton Mobility Index (DEMMI) at baseline, 8 weeks, and at 3-month follow-up

The DEMMI [9] is a widely used and psychometrically validated measure of mobility. Its 15 items assess a wide range of physical abilities, including sit unsupported in a chair, sit to stand from chair, stand on toes, walking distance, pick up pen from floor, and jump.

### Procedure

Volunteers attended two half-day training workshops and completed their baseline questionnaires before commencing their first partnership with a resident. A member of the research team visited resident participants individually to complete baseline questionnaires. Volunteers were then allocated a resident participant and commenced the intervention. Post-intervention and 3-month follow-up questionnaire data were collected from residents and volunteers on site at the participating aged care facilities. Volunteers who were unable to complete these questionnaires on site were mailed a questionnaire and a reply-paid envelope.

### Statistical analysis

Feasibility was assessed by determining if the a priori feasibility criteria described above were met. Given that depression was the primary psychological outcome variable of interest, reliable and clinically significant change in resident depressive symptoms was computed using the reliable change index (RCI), with an RCI value > 1.96 indicating reliable change [2]. For clinical significance, the PHQ-9 cutoff scores of 5, 10, 15 and 20 were used representing mild, moderate, moderately severe and severe depression, respectively [20]. We also computed standardized change scores using Hedges’ *g* together with its 95% confidence interval for all well-being outcomes. Hedges’ *g* is a derivative of Cohen’s *d* that corrects for upwards bias [33]. Following standard protocol, *g* values of 0.2, 0.5 and 0.8 were taken to indicate a small, medium and large effect, respectively [7]. As feasibility studies are typically underpowered to detect statistically significant effects, power analyses were conducted using *GPower* for non-significant treatment effects to determine the sample size needed to detect a significant change in psychological outcomes of depression, anxiety and flourishing.

## Results

### Demographics

Participant characteristics of those who commenced the intervention (resident *n* = 18; volunteer *n* = 13) are presented in Table 3. The mean age of residents was 84.6 (age range, 70–97), and the mean age of volunteers was 65.7 (age range, 43–85). The majority of both residents (*n* = 15, 83%) and volunteers (*n* = 10, 77%) were female. Participants in this study were ethnically diverse, born in nine countries from around the world.

**Table 3:** Participant characteristics

Outcome	Residents		Volunteers	
	*N*	Mean (SD)/%	*N*	Mean (SD)/%
Age	18	84.61 (6.57)	13	65.77 (10.57)
Gender				
Female	15	83%	10	77%
Male	2	11%	3	23%
Undisclosed	1	6%	0	0%
Education				
Up to primary school	8	44%	0	0%
Up to secondary school	6	33.3%	5	38%
Apprenticeship or diploma	3	17%	4	31%
University degree	1	6%	4	31%
Country of birth				
Australia	11	61%	7	54%
England	2	11%	2	15%
India	1	5.6%	1	8%
Philippines	1	5.6%	1	8%
Singapore	1	5.6%	1	8%
South Africa	1	5.6%	1	8%
Vietnam	1	5.6%	0	0%
Relationship status				
Married/de facto relationship	2	11%	9	69%
Single	16	89%	4	31%
Paid employment				
Yes	NA	NA	2	15%
No			12	85%
Volunteer status				
Engaged in regular volunteering	NA	NA	8	61.5%
No current volunteering outside the study			5	38.5%

### Primary outcome: Feasibility

#### Recruitment and participants

As seen in the study flowchart in Fig. 1, 36 prospective participants were identified by the aged care facility as being suitable for the study during the 6-month recruitment window. Of these, three (8%) were excluded due to the presence of severe cognitive impairment. The remaining 33 were approached by a member of the research team. Nine participants were not interested, of whom seven (78%) were male and two (22%) were female. The remaining 24 (67%) prospective participants were interested and gave informed consent. Of those who gave consent, four were ineligible with a PHQ-9 score less than five, and two withdrew (one due to severe anxiety and the other due to family request) before intervention commencement. A total of 18 residents commenced the intervention, demonstrating feasible resident interest and recruitment within the expected 6-month timeframe.

For the volunteers, nine people expressed an interest from the aged care provider volunteer database and seven (78%) enrolled in the study. A further 12 prospective volunteers expressed interest from the NARI volunteer database, and five (42%) enrolled in the study. One volunteer self-referred via word of mouth at her local church.

#### Acceptability

Residents reported being highly satisfied with the intervention, with a mean satisfaction rating of 6.93/8 (SD = 1.87). The vast majority (15/16) of those who commenced the programme reported they would recommend the programme to others. In qualitative feedback, 11 residents identified that having a ‘regular visitor’ was the part of the intervention that they liked the most. The majority of residents noticed positive changes in themselves over the course of the intervention, with five noticing an increased focus on pleasant and meaningful activity e.g., “I’m consciously thinking about what I’m doing and why I’m making sure that I do things. It’s good.” (Resident ID 2). Residents reported appreciating re-engagement with activities including exercise (Resident ID 5), bingo (Resident ID 12) and gardening (Resident ID 13). Post-intervention, two residents identified that declining physical health unrelated to the programme was a negative change that had affected their well-being over the course of the intervention.

Volunteers reported being highly satisfied with the programme, with a mean rating of 7.0/8 (SD = 1.53). All volunteers reported that they would recommend the programme to others. In qualitative feedback, the majority of volunteers (10) reported that the opportunity to develop a relationship with the resident was the highlight of the programme. Volunteers reported a range of positive changes in themselves during the intervention, including greater insight into the experience of living in residential care (*n* = 3), being a better listener (*n* = 2) and increased self-confidence in their ability to help others (*n* = 2). Volunteers also reported other benefits, such as gladness at the chance to help (Volunteer ID 3), empathy (Volunteer ID 5) and more awareness of ‘the values that really matter’ in life (Volunteer ID 4).

#### Attrition

The overall intervention completion rate for residents and volunteers was 87%. A total of 14/18 (78%) of residents completed the intervention, which is slightly lower than the 85% completion rate that was anticipated. Three residents withdrew during the intervention, and one resident died. Reasons for resident withdrawal included not wanting to be involved with activities (Resident ID 14) and being ‘involved enough with activities’ and not needing the additional support (Resident ID 18). There were no volunteer dropouts during the intervention, and two were partnered with two residents. Between post-intervention and follow-up, one resident died and a further four were lost to follow-up. As seven volunteers were lost to 3-month follow-up, there was insufficient data (*n* = 5) to report this timepoint for volunteers at the group level.

#### Safety

No adverse psychological events were reported during this study. One participant died due to unrelated reasons during the intervention, and one participant died between post-intervention and the 3-month follow-up period.

#### Data collection

Of the four residents who did not complete the intervention, post-intervention data was collected from two (50%); one was lost to follow-up and one had died.

Among the completers, rates of missing data were very low (2.5% for residents and 1.7% for volunteers), indicating feasibility of data collection. Post-intervention data from one resident completer went missing and so is not reported in the results.

#### Treatment fidelity

Eight out of 14 (57%) of resident-volunteer dyads who completed the intervention submitted evidence of activity scheduling, through use of activity scheduling sheets or written notes documenting activity plans. Thus, we found evidence of moderate treatment fidelity.

### Secondary outcome: Pre-post changes in well-being

#### Resident well-being

**Fig. 2:**
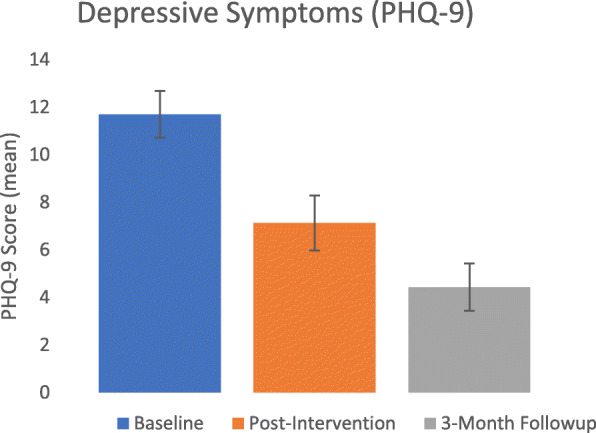
Mean resident depressive symptoms at baseline, post-intervention and 3-month follow-up

**Fig. 3:**
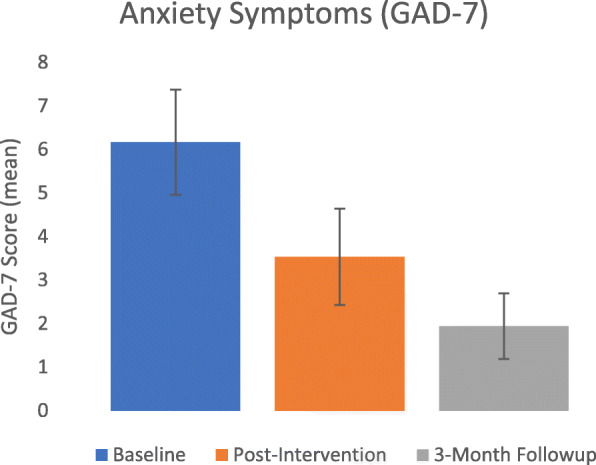
Mean resident symptoms of anxiety at baseline, post-intervention and 3-month follow-up

As seen in Figs. 2 and 3, the intervention was associated with improved resident well-being, with gains maintained—and improved—at follow-up.

Participation in the programme was associated with a large, statistically significant reduction in depressive symptoms, *Hedges’ g* = 1.14, *p* < 0.01, with the effect extending to *g* = 2.82 when comparing baseline to 3-month follow-up. Clinically, depression dropped from moderate severity pre-intervention (11.70) to subclinical symptoms at 3-month follow-up (4.43).

In terms of inter-individual variance in response, 11 residents had reliable reductions in depressive symptoms from pre- to post-intervention (including two non-completers), four residents had no reliable change (including one non-completer) and one resident had a reliable increase in depression.

There was also a significant reduction in anxiety from mild symptoms at baseline mean = 6.17 (5.12) to the subclinical range post-intervention, mean = 3.53 (4.29) (*g* = 0.61, *p* = 0.03). There was further decline when comparing baseline to follow-up, mean = 1.94 (2.12) (*g* = 1.35, *p* < 0.01).

In addition to a reduction in affective symptoms, there was also a non-significant trend towards increased positive well-being post-intervention (*g* = 0.53, *p* = 0.067). A power analysis revealed that a sample size of 23 would be needed to detect a significant effect, with 0.80 power. When comparing baseline to 3-month follow-up, the effect was significant (*g* = 0.92, *p* = 0.036).

#### Mobility

There was no significant change in resident mobility scores from baseline mean = 34.17 (15.89) to post-intervention mean = 32.25 (16.47), *t* (11) = .917, *p* = .38. Mobility levels also remained stable at follow-up mean = 34.62 (15.02).

#### Volunteer well-being

The intervention was associated with a non-significant increase in depressive symptoms (*g* = 0.38, *p* = 0.20) and a trivial and non-significant increase in anxiety (*g* = 0.05, *p* = 0.85). There was also a trivial and non-significant increase in positive well-being (*g* = 0.003, *p* = .99).

## Discussion

This volunteer-led behavioural activation intervention was found to be acceptable and feasible when delivered to depressed older adults residing in two residential aged care facilities. Both residents and volunteers were highly satisfied with the program, with a mean satisfaction rating of 7/8. Furthermore, all volunteers, and 94% (15/16) of residents reported that they would recommend the programme to a friend. Recruitment occurred within the expected 6-month timeframe, and there were reasonable intervention completion rates for both residents (78%) and volunteers (100%). Importantly, the intervention was associated with a significant and clinically meaningful decline in resident depressive symptoms, with remission to subclinical symptomatology achieved by 3-month follow-up. Volunteers had high mental health at baseline, so no quantitative changes in volunteer well-being were observed over the program, likely due to ceiling effects. However, qualitative feedback indicated that volunteers found their participation to be enjoyable and meaningful.

Importantly, we found evidence that the intervention was efficacious in reducing resident symptoms of both depression and anxiety, and these improvements were augmented at follow-up. Prior work shows that depression does not typically spontaneously remit in aged care settings [35, 38]. Whilst a controlled trial is needed to validate our results, given the chronic nature of depression in residential aged care, it is unlikely that observed improvements were due to chance alone. A recent meta-analysis found that befriending, a similar but less structured intervention commonly used in residential aged care, had no significant effect on depressive symptoms [34]. Thus, behavioural activation is a promising alternative that could have a substantial lasting impact to reduce depression and anxiety in residential care. Given the high comorbidity of depression and anxiety in older adults [26], it would make sense to consider including participants with both anxiety and depression in future trials.

This study adds to a growing body of literature demonstrating the applicability of behavioural activation in diverse settings where traditional psychological treatments of depression such as CBT may be less suitable [17] or prohibitive due to cost [31, 32]. A novel aspect of this study is that the behavioural activation intervention was delivered by trained volunteers, whereas prior behavioural activation interventions have primarily been delivered by specialist or non-specialist healthcare professionals [13]. Together with cost efficiency, an advantage of relying on trained volunteers is the potential for mutual benefits to both depressed residents *and* volunteer interventionists [28]. Indeed, in qualitative feedback, the volunteers reported substantial benefits from participating in the programme, including a sense of purpose and meaning. Since quantitative measures can be difficult to capture change in volunteer well-being due to ceiling effects, future work in the area should continue to measure the qualitative experiences and benefits of volunteering, in addition to quantitative outcomes. Potential long-term volunteer outcomes (e.g. morbidity and longevity) should also be considered [14].

Two challenges with a volunteer-led intervention relate to scalability and treatment fidelity. First, a volunteer-led intervention requires sufficient access to volunteers who are willing and able to commit to a relatively intensive intervention associated with 8 h of training and 8 weeks delivering the intervention, plus paperwork (e.g. supporting the resident to complete their weekly activity schedules) and phone call liaisons with a behavioural activation specialist. Whilst volunteer availability could be a problem, in this study, we drew on existing volunteer pools. Current aged care volunteers typically engage in relatively unstructured contact with residents. Drawing on these existing volunteer pools, there could be scope to introduce more structured interactions between volunteers and residents, for instance through the current behavioural activation intervention, that specifically target residents with depressive symptoms. Thus, this process is scalable and could be a powerful way to improve the impact of existing aged care volunteer programmes.

The second issue with a volunteer-led programme is ensuring treatment fidelity. In this study, we collected completed activity scheduling sheets from volunteers—to assess the extent to which they had discussed and scheduled pleasant and meaningful activities for the resident to participate in over the week ahead. Quite a large proportion of volunteers (42%) did not submit resident activity schedules. In the written qualitative feedback, three residents said that they found the activities scheduling to be unhelpful, with one elaborating that they ‘would rather be flexible’. Balancing activity planning with the need to be flexible is important. Exploring ways to get residents ‘on board’ with activity scheduling should also be integrated into future volunteer training programmes for this intervention. Treatment fidelity might be improved with more active monitoring of activity schedule use, recording some sessions, and refresher training during the intervention. Since volunteers indicated the desire for a flexible intervention, it would be important to integrate this feedback into the design of a future trial, whilst maintaining the core components of the intervention. This could be achieved through a co-design model whereby volunteers provide more extensive input to inform the structure and content of the intervention, as well as potentially enhanced volunteer training to ensure the volunteers understand and feel competent in delivering the core components of the behavioural activation intervention.

It is important to note that the majority of both residents (83%) and volunteers (77%) were female. Of the nine prospective participants who were approached but not interested in participating in the study, seven (78%) were male, and in total, only two male residents elected to enrol in the study. Further work to better understand men’s reasons for declining to participate and consideration of how to tailor the intervention to better suit the needs of men is needed.

## Conclusion

In sum, this study found that an 8-week volunteer-led behavioural activation intervention was feasible and acceptable to depressed older adults residing in aged care facilities. Furthermore, the intervention appeared to be efficacious in ameliorating both depression and anxiety, with gains maintained at 3-month follow-up. Given that depression is both extremely common and under-treated in aged care facilities, a larger randomized controlled trial is warranted to expand on these promising results.

## Data Availability

The datasets generated during the current study are not publicly available due to the ongoing nature of this research but are available from the corresponding author on reasonable request.
